# Supplementary material for: Utility of the ELISpot Test to Predict the Risk of Developing BK Polyomavirus Nephropathy in Kidney Recipients, a Multicenter Study
Source: Vaccines (Basel). 2025 Jul 28;13(8):796. doi: 10.3390/vaccines13080796 (PMC12390299; doi:10.3390/vaccines13080796)
Supplement: Supplementary file 1 [file vaccines-13-00796-s001.zip › vaccines-3730912-supplementary.pdf]

Table S1. Univariate analysis of main characteristics of kidney recipients according to study participation

	**Screened cohort**<br><br>**n=72**	**Final study population**<br><br>**n=320**	***p* value**
**Age, median of years (IQR)**	56.8 (13.6)	56 (13.2)	0.4
**Male sex, n (%)**	44 (61)	201 (63)	0.4
**Renopancreatic transplantation, n (%)**	7 (10)	32 (10)	1
**Chronic kidney disease etiology**			0.6
Diabetic nephropathy, n (%)	21 (29)	83 (26)	
Polycystic kidney disease, n (%)	5 (7)	35 (11)	
Nephroangiosclerosis , n (%)	4 (6)	22 (7)	
Obstructive uropathy, n (%)	3 (4)	16 (5)	
Other or undeterminated, n (%)	39 (54)	164 (51)	
**Deceased donation, n (%)**	62 (86)	262 (82)	0.7
**Induction,** n(%)	72 (100)	314 (98)	0.3
Antilymphocyte globulin	36 (50)	161 (50)	
Basiliximab	35 (49)	143 (45)	
**Immunosuppression regimen**			0.45
Calcineurin inhibitor-based regimen, n (%)	47 (65)	198 (62)	
mTOR-based regimen, n (%)	25 (35)	122 (38)	
